# 230. Nontyphoidal *Salmonella* from Clinical and Retail Meat Sources Reveal Antimicrobial Resistance Genes for Ceftriaxone and Ciprofloxacin

**DOI:** 10.1093/ofid/ofab466.432

**Published:** 2021-12-04

**Authors:** Nkuchia M M’ikanatha, Xin Yin, Yezhi Fu, Sameera Sayeed, Christopher Carr, Lisa Dettinger, Nicole Hackman, Edward Dudley, Heather Tate

**Affiliations:** 1 Pennsylvania Department of Health, Harrisburg, PA; 2 Penn State College of Medicine, Hershey, Pennsylvania; 3 Penn State University, State College, Pennsylvania; 4 Georgia Tech University, Atlanta, Pennsylvania; 5 Penn State Health, Hershey, Pennsylvania; 6 Food and Drug Administration, Laurel, MD

## Abstract

**Background:**

Pennsylvania participates in the National Antimicrobial Resistance Monitoring System (NARMS), which includes monitoring of Nontyphoidal *Salmonella* (NTS), a leading cause of bacterial foodborne illnesses in the United States.

**Methods:**

Clinical NTS isolates submitted to the Pennsylvania Department of Health (2015-18) were tested for susceptibility to 15 antimicrobial agents and analyzed by whole-genome sequencing (WGS). Concurrently, we conducted a prospective microbiological survey of NTS in retail meat products (chicken breasts, ground turkey, and pork chops) with susceptibility testing and WGS.

**Results:**

Of a sample of 426 clinical *Salmonella* isolates from humans analyzed for antimicrobial susceptibility, 65 (15.3%) had decreased susceptibility to ciprofloxacin (DSC). Ampicillin resistance was observed in 39 (9.2%) and 15 (3.5%) were ceftriaxone-resistant. Ten ceftriaxone-resistant isolates had genetic elements that confer resistance to third generation extended-spectrum cephalosporins (ESC) [*bla*_CMY−2,_ n=8 and *bla*_CTX-M-65,_ n=2]. The *bla*_CTX-M-65-_ positive isolates had a mutation in *gyrA* that confers fluoroquinolone resistance. Thirteen clinical isolates carried plasmid-mediated fluoroquinolone resistance genes (PMQR) [*qnrB19, qnrS1, qnrA1*]. We detected NTS in 131 (3.8%) of 3480 meat samples tested. 7 (5.3%) had DSC, while 38 (29%) and 21 (16%) were resistant to ampicillin and ceftriaxone, respectively. Four *S*. Infantis isolates had DSC and a *bla*_CTX-M-65_ gene plus a mutation in *gyrA*. Thirteen meat isolates had the *bla*_CMY-2_ gene. One additional *bla*_CTX-M-65-_positive S. Infantis without *gyrA* from ground turkey (SRR6351119) differed from four clinical isolates by ≤10 single-nucleotide polymorphisms. Percent of isolates from patients and meat sources that demonstrated resistance to amoxicillin-clavulanate (AMC), ceftriaxone, and decreased susceptibility to ciprofloxacin (DSC) to nine antimicrobial classes tested.

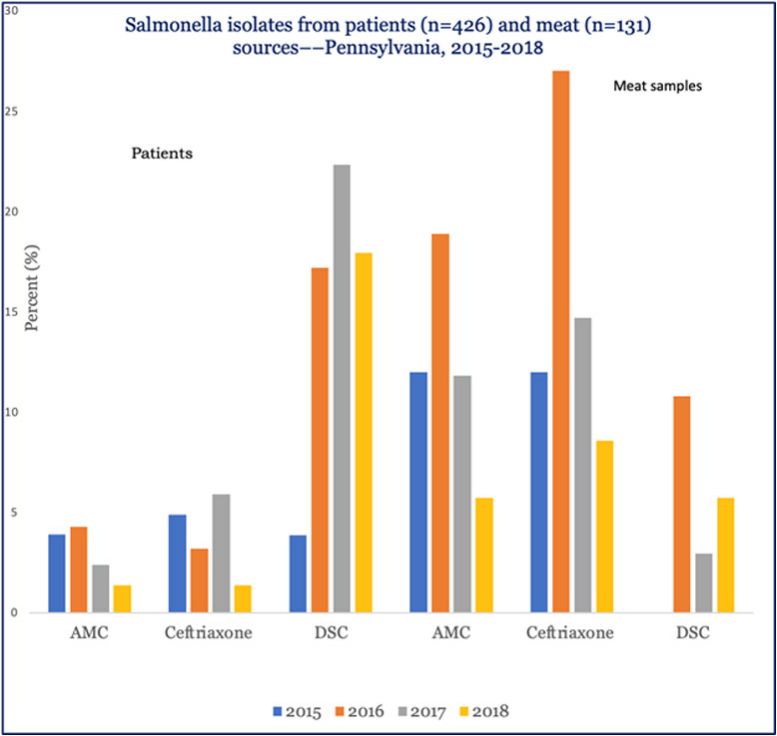

Among isolates from patients, resistance to ceftriaxone, a third-generation cephalosporin preferred for severe infections in children, increased from zero in 2015 to 5.8% in 2017. Overall, DSC increased in isolates from human sources while in strains from meat sources, DSC increased from zero in 2015 to over five percent in 2018.

**Conclusion:**

NTS isolated from human and meat sources were multi-drug resistant. Demonstration of similar resistance genes in meat and in ill humans may be consistent with spread of antibiotic-resistant pathogens from food sources. Dissemination of genes that confer resistance to third generation cephalosporins and fluoroquinolones, including some on mobile plasmids, may undermine recommended treatment for severe NTS infections. These results underscore the need for antimicrobial stewardship efforts in both agriculture and human medicine.

**Disclosures:**

**All Authors**: No reported disclosures

